# Psychological Impact of Stalking on Male and Female Health Care Professional Victims of Stalking and Domestic Violence

**DOI:** 10.3389/fpsyg.2018.00321

**Published:** 2018-03-13

**Authors:** Daniela Acquadro Maran, Antonella Varetto

**Affiliations:** ^1^Department of Psychology, University of Torino, Turin, Italy; ^2^A.O.U. Città della Salute e della Scienza di Torino, Turin, Italy

**Keywords:** anxiety, depression, distress, prevention, hospitals, gender differences, relationships

## Abstract

The aim of this work was to investigate stalking experiences in a sample of Health Care Professionals, or HCPs, who experienced domestic violence in their previous relationships with an intimate romantic or non-romantic who had become their stalkers. A comparison between males and females was made to highlight the differences among the genders. The findings showed that, for the most part, the victims experienced stalking by a stalker that was not of the same gender. Moreover, the nature of the relationship was romantic, for the most part, for both female and male subjects, suggesting that the principal motivation of stalking is the disruption of an intimate relationship. Regarding domestic violence, females described the phenomenon from a different perspective, indicating verbal, physical, and sexual abuse, while males indicated only verbal abuse. Females tended to amplify, more than the males, depression, and state and trait anxiety. Even if all symptoms were expressed in both females and males, the males exhibited a lack of confidence in their bodies, and the emotional literacy made the expression of distress more difficult. At the same time, the expression of anxiety presented in the women permitted them to become progressively less victimized over time; depression and anxiety allow the recognition of these symptoms as signs of distress and to intervene to reduce them.

## Introduction

Stalking has been defined by Westrup (1998) as a set of repetitive behaviors directed toward a target that perceives those behaviors as unwelcome and intrusive. As a consequence, victims of stalking experience fear for their safety (Sheridan et al., 2003) and/or for closure (Dennison, 2007). Since the 1990s, national surveys have been made in several countries (see for example Tjaden and Thoennes, 2000) using different criteria both to define the phenomenon (e.g., the duration of the stalking campaign), the method used (e.g., interview by phone or online survey), and the gender of the subject involved (female and/or male). This limitation did not allow the stalking campaign to be defined in a unique way, influencing (and being influenced – De Fazio, 2009) the boundaries of the phenomenon. Despite these limitations, Spitzberg and Cupach (2003, 2007) identified the prevalence rate of victimization among females and males in the general population. While the prevalence rate in females ranged from 8 to 32%, the range was 2–13% in males. Thus, the risk of victimization among females is higher, particularly in young individuals (18–29 years) and may extend to when the stalker tries to establish or reestablish a relationship. On average, the analysis of the literature by Spitzberg and Cupach (2007) highlighted a duration of 22 months, with a frequency of the contact that Sheridan and Davies (2001) found in their investigation to be ‘every day’ for more than one third of the victims.

Further research tried to understand the nature of the victim–stalker relationship and previous domestic violence (defined as “any violence between current or former partners in an intimate relationship, wherever and whenever the violence occurs,” Walby and Allen, 2004, p. 4), the behavior that characterized the stalking campaign, the physical and emotional consequences, and the coping strategies adopted. Regarding this relationship, Sheridan et al. (2003) described the stalker as an ex-intimate, an acquaintance (e.g., friend, relative, colleague, or patient) or a stranger. From their work, it appears that prior domestic violence increases the chances of becoming a victim of stalking.

Moreover, as underlined by Senkans et al. (2017), if the intimate relationship was characterized by violence, this will be perpetrated also during the stalking campaign; the end of the relationship is not the end of the abusive behavior (Palarea et al., 1999; Douglas and Dutton, 2001; Roberts, 2005). The behaviors that characterize the stalking campaign have been described as unwanted communication (any contact with the stalkers, such as telephone calls, e-mails, letters or cards, text messages), unwanted approaches (the stalker followed, damaged property, visited or waited outside home and/or workplace) (McEwan et al., 2012), harassment and intimidation (threats, asking for information, spreading lies) (Spitzberg and Cupach, 2014). In their investigation, in which 107 victims of stalking were involved, Ferreira and Matos (2013) found that the majority of participants reported they had experienced partner abuse before the beginning of the stalking campaign (caused by the end of the relationship). Among these, the findings revealed that stalking victims suffer from a very diverse spectrum of behaviors, more frequently unwanted communication and contact, while harassment and intimidation are the least frequent. The stalking campaign leads to physical and emotive consequences for the victims, described by Littel (1999) as ‘soul destroying.’ Among the physical consequences, Spitzberg and Cupach (2007, 2014) indicated e.g., forms of addictions, appetite, or sleep disturbances, while among the emotive consequences they suggested the examples of anger, confusion, and fear. Mullen et al. (2006) highlighted the importance of investigating depressive and anxious reactions because these symptoms are linked to the experience of stalking victimization (Dressing et al., 2005), in particular in victims of ex-partners (Davis and Frieze, 2000). To confirm this, findings from the investigation by Sheridan and Lyndon (2012), which involved 1,214 self-defined stalking victims, showed that victims who had a prior relationship with their stalkers were more likely to experience a greater number of emotive, physical, and social consequences than other types of victim–stalker relationships. To cope with the phenomenon, victims use several strategies. Spitzberg and Cupach (2014) categorized these coping strategies (see also Amar and Alexy, 2010) as the following:

(1) Moving toward involves trying to ‘reason with’ the stalker, to negotiate a different type of relationship (e.g., moving from a closer intimate relationship to a closer friendship) or asking the stalker to stop their campaign (e.g., reasoning with the stalker);
(2) Moving away involves the avoidance and limitation of the access of the stalker (e.g., ignoring the stalker);
(3) Moving against involves threatening, harming, or otherwise deterring the stalker (e.g., threatening him/her verbally);
(4) Moving inward involves “any effort or acts to repair, empower, enrich, or merely focus on self as the source of managing the disruption of unwanted pursuit, independent of others role in the episode” (Cupach and Spitzberg, 2004, p. 145). (e.g., self-defense class);
(5) Moving outward involves seeking help, guidance, and assistance (e.g., told a friend, relative, or neighbor).

Cupach and Spitzberg (2004) highlighted that victims engage in multiple strategies to cope with stalking campaigns. Ferreira and Matos (2013) highlighted that the most used coping strategies by victims of stalking, previously victims of domestic violence, were to search for help from friends or family (moving outward, following the categorization of the coping strategies suggested by Spitzberg and Cupach), to negotiate with and to confront the stalker (moving toward), and to avoid the stalker (moving away).

An analysis of the literature revealed that one of the samples most investigated was HCPs (see McIvor et al., 2008). Findings from investigations involving psychiatrists, physicians, nurses, psychologists, and among others have shown that this population is at higher risk of victimization than the general population (Ashmore et al., 2006; Abrams and Robinson, 2011; Whyte et al., 2011; Paraschakis and Konstantinidou, 2012; Mastronardi et al., 2013). In their literature analysis, Spitzberg and Cupach (2003) argued that the average incidence was 13.9% for samples from ordinary populations, while the prevalence rate of victimization in HCPs ranged from 12 to 50% (Acquadro Maran et al., 2014a). In this population, the risk of victimization has been linked to both to the nature of their work and to the expectation about the engagement in the relationship (Galeazzi et al., 2005). HCPs have close contact with people suffering from physical and/or mental disease. Their professional behavior, involving being devoted to caring for the patient, could be misunderstood as a desire to begin a relationship (intimate romantic or non-romantic). The motion to change the nature of the relationship from the HCP could cause disillusioned beliefs, feelings of frustration, desires of revenge, and so on (Galeazzi et al., 2005). Thus, a stalking campaign begins with the aim of establishing a relationship. On the other hand, people (partners, friends, and relatives) have expectations about the quality of the care that HCPs provide, also outside the workplace. These expectations are linked to the attention to the relationship and, as has emerged in previous research (Acquadro Maran et al., 2017), the failure to fulfill it could trigger emotive reactions such as anger and jealousy. In this case, the stalking campaign could begin with the desire of revenge.

A gap in the literature is related to the experience of domestic violence in this population and the experience of stalking victimization. An analysis of the literature showed that HCPs are asked to prevent and/or to intervene in domestic violence (see e.g., Krug et al., 2002). The possibility that they could themselves be victims of domestic violence was not considered. However, previous investigations showed that HCPs are victims by acquaintances and ex-partners (Ashmore et al., 2006; Whyte et al., 2011).

### Current Study

The aim of this work was to investigate stalking experiences in a sample of Italian female and male HCPs who experienced domestic violence and stalking victimization. The Italian context has been characterized since 2009 by the introduction of the anti-stalking law (Penal Code, article 612 *bis*, 2009). This law states that: “Provided the act is not recognized as a more serious crime, it is a criminal offence, punishable with imprisonment ranging from 6 months up to 4 years, to continuously threaten or harass another person to such an extent as to cause a serious, continual state of anxiety or fear, or to instill in the victim(s) a motivated fear for his/her own safety or for the safety of relatives or other persons linked to the victim(s) by virtue of kinship or emotional relationship or to force the victim(s) to change his/her living habits.”. Previous research on Italian HCP victims of stalking, showed that among 107 nurses, 28 were victims of an ex-partner, while 44 were victims of acquaintances (Acquadro Maran et al., 2014a). In an investigation among 256 HCPs, it emerged that stalkers were ex-partners of 88 victims, while for 95 they were acquaintances (Acquadro Maran et al., 2017).

The general goal of our study was to evaluate the stalking experience in a sample of HCPs that experienced domestic violence in their previous relationship with an intimate romantic or non-romantic that became their stalkers. A comparison between males and females was made to highlight the differences between the sexes. The variables investigated were those described by the literature (see above): nature of the relationship, stalking behaviors, the frequency and the duration of the stalking campaign (one item each), the consequences, and the coping strategies used. Given the lack of literature on this topic, we did not have specific hypotheses about the gender difference in the stalking experience of females and males who experienced domestic violence.

The data were gathered from a survey on stalking victimization involving more than 4000 HCP who worked in 6 Italian hospitals (public sector), and 1901 questionnaires were filled out (47.2%). The HCPs self-declared victims of stalker were 272 (14.3%). A selection was made among the cases of self-declared victimization. The criteria of inclusion were the nature of the relationship [intimate romantic (such as partner and ex-partner) and intimate non-romantic (relatives, close friends – see Spitzberg, 2002)] and the presence of a form of domestic violence (verbal abuse, physical harm, sexual abuse – Bennett Cattaneo et al., 2011). Victims of other types of stalkers (acquaintance, unknown) and those who did not suffer from domestic violence were not included.

## Materials and Methods

### Participants

In accordance with the above-mentioned criteria, and among more than 270 cases of self-declared victimization (205 female, 67 male), 147 (54%) were selected. The participants were aged 19–60 years (mean age 36 years, *SD* = 11.24), and most of them 96 (65.3%) were female. Overall, most of them were nurses (59, 40.1%), psychologists, (37, 25.2%), physicians (22, 15%), health technicians (14, 9.5%), and health care operators (9, 6.1%). Six (4.1%) HCPs did not answer this question. About a quarter were single (36, 24.5%), 35 participants were engaged (23.8%), 35 were married (23.8%), 18 were cohabiting (12.2%), 18 were divorced (12.2%), and one was widowed (0.7%). The remainder of the sample (4, 2.7%) did not answer this question. The stalker was in most cases a male (101, 68.7%), and he/she was aged 17–80 (*M* = 35.8, *SD* = 12.47). In 13 (8.8%) cases, the stalker was unemployed. All victims experienced verbal abuse before the beginning of the stalking campaign, 15 (10.2%) experienced physical harms, and 4 (2.7%) experienced sexual abuse. All respondents took part on a voluntary basis.

### Materials

Participants were asked to anonymously complete several sections of a self-administered questionnaire. The first section described the purpose of the questionnaire and contained the instructions for replying, as well as the anonymity and privacy statements. The modified Italian version of the questionnaire constructed by the Network for Surviving Stalking (NSS) with Dr. Lorraine Sheridan (Forensic Psychologist, University of Leicester), a questionnaire on depression, and two scales on anxiety were used to describe the experience of victimization. The Italian version of the stalking questionnaire covered issues such as demographic details of the participants and the stalkers and the duration and frequency of stalking. These were followed by yes/no type questions about the following:

(1) the nature of their relationship (intimate romantic or non-romantic – 2 items, Cronbach’s α = 0.64);
(2) the presence of domestic violence (verbal abuse, physical harm, sexual abuse; 3 items, Cronbach’s α = 0.62);
(3) the stalking behaviors (e.g., ‘the stalker threatened me with physical violence’; 14 items, Cronbach’s α = 0.68);
(4) the frequency and duration of the stalking campaign (one item each);
(5) the physical and emotional consequences (e.g., ‘nausea’ and ‘fear,’ respectively) (19 items; Cronbach’s α = 0.74);
(6) the coping strategies used (e.g., ‘did you talk with your partner/friend/relative about the stalking campaign?’; 16 items, Cronbach’s α = 0.62).

The coping strategies were subsequently categorized as suggested by Spitzberg (2002) and Spitzberg and Cupach (2007) as moving toward, moving away, moving against, moving inward, or moving outward (e.g., told friend, relative, or neighbor).

The Beck Depression Inventory (BDI, Beck et al., 1961; Italian version by Scilligo, 1988) and the State-Trait Inventory (STAI, Spielberger, 1983; Italian version by Pedrabissi and Santinello, 1989) were used to investigate the psychological consequences of the victimization in each stalking campaign. The BDI is a 21-question survey designed to determine the presence of depression symptoms. Scoring permits the classification of depression as minimal (scores 0–13), mild (14–19), moderate (29–28), and severe depression (> 29) (in this study Cronbach’s α = 0.96). The STAI consists of two forms to measure the state (Y1 form, how the victim of stalking feels ‘right now,’ at this moment) and trait (Y2 form, how the stalking victims feel most of the time) anxiety. Each scale includes 20 items. The total scores can range between 20 and 80, where 40 is the threshold value considered predictive of anxiety symptoms. A rating scale defines the level of severity, with 40–50 indicating mild, 50–60 indicating moderate, and >60 indicating severe anxiety. Cronbach’s α was 0.87 and 0.86, respectively. All the questionnaires were self-administered.

### Procedure

A letter with the invitation to take part in the investigation on HCPs victims of stalking was sent out to six hospitals. In the letter, we explained the purpose of the research, the voluntary nature of participation, the anonymity and privacy statement in accordance with Italian Law and with the Declaration of Helsinki, the scales that would be used and the procedure for completing and collecting the questionnaires. Hospital administrations and local guarantee committees evaluated, endorsed, and authorized the research, allowing researchers to use the data for scientific purposes. Upon approval, Department Chiefs from each unit/service were asked for authorization to administer the questionnaire to the HCPs and to define the method of delivery of the questionnaires.

Each participant was given a printout of the questionnaire, the information letter, and the informed consent form in accordance with the Declaration of Helsinki. The first page of the questionnaire contained the aim of the research, the instructions for completing and returning the questionnaires and the contact details of the researchers (the authors of this paper) for any doubts or problems. The stalking phenomenon was described on the first page. The definition by Galeazzi and Curci (2001), similar to that set forth in article 612 *bis*, 2009 of the Italian Penal Code, was used: “a repetitive pattern of behavior, intrusive surveillance and control, unwanted communication or contact with a victim, which causes a state of fear and/or anxiety and/or annoyance (for the victim him- or herself and/or for his or her loved ones)” (Acquadro Maran et al., 2017, p. 2608).

All HCPs were asked to complete the first part of the questionnaire (socio-personal data). After this section, one question discriminated victims and non-victims: “referring to the previous description of the phenomenon, have you been a victim of stalking during the lifetime?.” Consequently, for those subjects that self-declared as a victim of stalking, the questionnaire continued onto the next sections. For those who answered ‘no,’ the questionnaire ended. For all, the request was to place the questionnaire in a sealed box situated in the locker room. The scheduled date for collection was after a 3-week period (after 1 week there was a reminder placed on the sealed box).

### Data Analysis

The data were processed using SPSS version 24 to produce mainly descriptive and inferential statistics. Descriptive measures (means ± SD) were calculated for all test variables for the two groups of victims (male, female). The test of Chi-square (χ^2^) was used to measure the differences between groups in terms of the categorial variables (sex male/female, yes/no answer to the stalking questionnaire) and the different cut-offs that indicated the level of depression and state and trait anxiety symptoms (minimal, mild, moderate, or severe). Differences were considered statistically significant if *p* < 0.05. Correlations were calculated to examine the relation between the number of physical and emotional symptoms reported by female and male victims of stalking and depression and anxiety symptoms, and between the number of methods of harassment and the coping strategies used by victims (female, male).

## Results

### Female HCPs Victims of Stalking

Female HCPs who self-declared as victims of domestic violence and stalking were 96 (65.3–46.8% of victims among the female HCPs victims of stalking), aged 19–60 years (*M* = 35, *SD* = 11.40). Most of them were nurses (38, 39.6%), psychologists, (27, 28.1%), physicians (15, 15.6%), health technicians (8, 8.3%), and health care operators (7, 7.3%). About one third were married (27, 28.1%), 27 participants were engaged (28.1%), 19 were single (19.8%), 12 were cohabiting (12.5%), 10 were divorced (10.4%), and one did not give an answer.

The stalker was in most cases a male (88, 91.7%). He/she was aged 17–80 years (*M* = 35.2, *SD* = 12.54). The stalker was an employee in most cases (83, 86.4%), and 25 (26%) were HCPs. The nature of the relationship with the victims was intimate romantic in 67 cases (69.8%) and intimate non-romantic in 31 (32.3%) cases. The domestic violence was described by the victims as verbal abuse (all respondents), physical harm (15, 15.6%), and sexual abuse (3.1%). The behaviors that characterized the stalking victimization are in **Table 1**. Among ‘others’ behaviors, one female described ‘knocking at the window.’ On average, females experienced five different behaviors, and in most cases, the victims (59, 61.5%) affirmed that the frequency of the behaviors was ‘every day.’ A total of 10 victims (10.4%) declared that they were still victims, 13 (13.5%) did not know, and the rest (73, 76%) said no. The duration of the stalking campaign was on average, more than 1 year (range 2–120 months, *M* = 16.57, *SD* = 26.11).

**Table 1:** Behaviors characterizing the male and female HCPs experience of stalking victimization (*N* = 147).

	Male	Female	χ^2^	*p*
	*n* = 51	*n* = 96
Acts of vandalism	5 (9.8)	21 (21.9)	1.39	n.s.
Asking for information	16 (31.4)	32 (33.3)	1.44	n.s.
Following	17 (33.3)	47 (49)	0.09	n.s.
Sending gift	5 (9.8)	11 (11.5)	0.19	n.s.
Sending e-mail, letters, or cards	33 (64.7)	52 (54.2)	5.57	0.019
Spreading lies	10 (19.6)	30 (31.3)	0.13	n.s.
Text message	18 (35.3)	40 (41.7)	0.46	n.s.
Telephone calls	24 (47.1)	61 (63.5)	0.14	n.s.
Threats	7 (13.7)	26 (27.1)	0.87	n.s.
Visiting home	3 (5.9)	12 (12.5)	0.39	n.s.
Visiting workplace	10 (19.6)	30 (31.3)	0.18	n.s.
Waiting outside home	11 (21.6)	46 (47.9)	2.44	n.s.
Waiting outside workplace	12 (23.5)	36 (37.5)	0.36	n.s.
Other	5 (9.8)	7 (7.3)	1.54	n.s.


*Percentage values are given in parentheses. χ^*2*^ = chi-square; *p* = *p*-values; n.s. = not statistically significant.*

The stalking campaign left the victims with both physical and emotive consequences (see **Table 2**). Female victims of stalking suffered from 1 to 4 different physical consequences (*M* = 1.23, *SD* = 0.60) and from 1 to 3 different emotive consequences (*M* = 1.21, *SD* = 0.49). Regarding depression and anxiety, the results showed that, for the most part, for females the level of depression was minimal (**Table 3**), even if the females experienced symptoms more often than males. Regarding trait anxiety, the findings showed that females were more prone than males to reach the cut-off for moderate anxiety (STAI-Y2). The number of emotive consequence was significantly related to the increase in depression symptoms (**Table 4**). To cope with the stalking campaign, the victims adopted different strategies (**Table 5**). All victims adopted at least one strategy of the moving away type. Inferential statistics showed that when the number of stalkers’ behaviors increased, the use of the following coping strategies decreased: moving away, moving against, moving inward, and moving outward (**Table 6**).

**Table 2:** Physical and emotional symptoms characterizing the male and female HCPs experience of stalking and domestic violence victimization (*N* = 147).

	Male	Female	χ^2^	*p*
	*n* = 51	*n* = 96
**Physical symptoms**				
Weight change	5 (9.8)	21 (21.9)	0.88	n.s.
Stomach trouble	5 (9.8)	22 (22.9)	0.89	n.s.
Sleep disorder	19 (37.3)	46 (47.9)	0.25	n.s.
Headache	10 (19.6)	30 (31.3)	0.03	n.s.
Weakness	8 (15.7)	25 (26)	0.04	n.s.
Nausea	4 (7.8)	9 (9.4)	0.20	n.s.
Panic attacks	7 (13.7)	22 (22.9)	0.11	n.s.
**Emotional symptoms**				
Suicidal thoughts	0 (0)	4 (4.2)	1.43	n.s.
Sadness	4 (7.8)	11 (11.5)	0.02	n.s.
Apprehension	27 (52.9)	51 (53.1)	2.48	n.s.
Anger	28 (54.9)	46 (47.9)	4.73	0.023
Fear	17 (33.3)	50 (52.1)	0.48	n.s.
Lack of confidence	2 (3.9)	16 (16.7)	2.13	n.s.
Aggressiveness	6 (11.8)	12 (12.5)	0.48	n.s.
Paranoia	8 (15.7)	18 (18.8)	0.57	n.s.
Confusion	9 (17.6)	29 (30.2)	0.08	n.s.
Irritation	14 (27.5)	30 (31.3)	0.68	n.s.
Agoraphobia	3 (5.9)	4 (4.2)	1.15	n.s.


*Percentage values are given in parentheses. χ^*2*^ = chi-square; *p* = *p*-values; n.s. = not statistically significant.*

**Table 3:** Level of depressive and anxiety symptoms indicated by the male and female HCPs experiencing stalking and domestic violence victimization (*N* = 147).

	Male	Female	χ^2^	*p*
	*n* = 51	*n* = 96
BDI:				
- minimal	40 (78.4)	75 (78.1)	0.01	n.s.
- mild	5 (9.8)	10 (10.4)	0.01	n.s.
- moderate	2 (3.9)	8 (8.3)	0.81	n.s.
- severe	4 (7.8)	3 (3.1)	0.88	n.s.
STAI Y1:				
- minimal	26 (51)	29 (30.2)	4.18	0.036
- mild	19 (37.3)	53 (55.2)	2.42	n.s.
- moderate	4 (7.8)	10 (10.4)	0.30	n.s.
- severe	2 (3.9)	4 (4.2)	0.05	n.s.
STAI Y2:				
- minimal	26 (51)	18 (18.7)	11.14	0.001
- mild	21 (41.2)	55 (57.3)	2.12	n.s.
- moderate	2 (3.9)	19 (19.8)	4.23	0.033
- severe	2 (3.9)	4 (4.2)	0.17	n.s.


*Percentage values are given in parentheses. χ^*2*^ = chi-square; *p* = *p*-values; n.s. = not statistically significant.*

**Table 4:** Correlation between the number of physical and emotional symptoms reported by male and female HCP victims of stalking and domestic violence and depressive and anxiety symptoms (*N* = 147).

	Male		Female	
	*n* = 51		*n* = 96	
		
	Physical	Emotive	Physical	Emotive
BDI	0.19	0.18	0.23	0.32^∗^
STAI Y1	0.58^∗∗^	0.36	0.07	0.02
STAI Y2	0.51^∗∗^	0.38	0.03	0.02


*^∗^ = *p* < 0.005; ^∗∗^ = *p* < 0.010.*

**Table 5:** Typology of coping strategies used by male and female HCP victims of stalking and domestic violence (*N* = 147).

	Male	Female	χ^2^	*p*
	*n* = 51	*n* = 96
Moving toward	9 (17.6)	4 (4.2)	6.90	n.s.
Moving away	51 (100)	96 (100)	0.45	n.s.
Moving against	25 (49)	56 (58.3)	8.57	n.s.
Moving inward	38 (74.5)	76 (79.2)	0.93	n.s.
Moving outward	25 (49)	51 (53)	2.04	n.s.


*Percentage values are given in parentheses. χ^*2*^ = chi-square; *p* = *p*-values; n.s. = not statistically significant.*

**Table 6:** Correlation between the number of methods of harassment and the coping strategies used by male and female HCP victims of stalking and domestic violence (*N* = 147).

	Male	Female
	*n* = 51	*n* = 96
Moving toward	-0.56	-0.40
Moving away	-0.43^∗∗^	-0.30^∗∗^
Moving against	-0.57^∗∗^	-0.28^∗^
Moving inward	-0.00	-0.39^∗∗^
Moving outward	-0.02	-0.34^∗^


*^∗^ = *p* < 0.005; ^∗∗^ = *p* < 0.010.*

### Male HCPs Victims of Stalking

Male HCPs who self-declared as victims of stalking were (51, 34.7–76.1% of victims among the male HCPs victims of stalking), aged 21–60 years (*M* = 38, *SD* = 11.49). Most of them were nurses (21, 41.2%), psychologists (10, 19.6%), physicians (7, 13.7%), health technicians (7, 13.7%), and one (2%) health care operator. Three HCPs did not give any information about their work. More than one third were single (17, 33.3%), eight participants were engaged (15.7%), eight were married, eight were divorced, six (11.8%) were cohabiting (12.5%), and one was a widower. Three participants did not give an answer to this question.

The stalker was in most cases a female (34, 66.7%). He/she was aged 20–65 years (*M* = 37.07, *SD* = 12.38). The stalker was an employee in most of the cases (37, 73.5%), and 16 (31.3%) were HCPs. The nature of the relationship with the victims was intimate romantic in 37 (72.5%) cases, while in 14 cases (27.4%) it was intimate non-romantic. The domestic violence was described by all victims as verbal abuse. None of the male respondents indicated physical harm or sexual abuse. The stalking campaign was characterized by different behaviors (see **Table 1**). Among ‘others’ behaviors, the participants described the ‘the stalker threatened self-injury.’ On average, males experienced three different behaviors. Half of the victims (26, 51%) affirmed that the frequency of the behaviors was ‘every day.’ A total of 6 victims (11.8%) declared that they were still victims, 10 (19.6%) did not know, and the rest (35, 68.6%) answered no. The durations of the stalking campaigns were similar to those of the females, but it was a little longer, on average being more than 1 year, with a range of 2–132 months (*M* = 16.65, *SD* = 22.69). Male victims suffered, more than females, from the unwanted written communication method of harassment (“sending e-mails, letters, or cards”). The physical and emotive consequences that characterized the stalking campaigns are in **Table 2**. Male victims suffered from 1 to 3 different physical consequences (*M* = 1.07, *SD* = 0.34) and at least one emotive consequence (*M* = 1.05, *SD* = 0.35). The males suffered, more than the females, from ‘anger.’ The results showed that for the most part, for the males, the levels of depression, state trait, and anxiety were minimal (**Table 3**) and that they were less prone than females to experience these symptoms. The physical consequence was significantly related to both state and trait anxiety symptoms (**Table 4**). To cope with the stalking campaign, victims adopted different strategies (**Table 5**). Similar to the sample of females, all the males adopted at least one strategy of the moving against type. The stalkers’ use of different behaviors was significantly related with the decrease in the use of the moving away and moving against coping strategies (**Table 6**).

## Discussion

The aim of this work was to compare female and male HCP victims of domestic violence and stalking. The findings showed that, for the most part, the victims experienced stalking by a stalker who was not of the same gender, confirming that the phenomenon is most frequently inter-gender, particularly when the victim was a female (Sheridan et al., 2016). Moreover, the nature of the relationship was romantic for the most part for victims, both female and male, suggesting that the principal motivation of stalking was the disruption of an intimate relationship (Tassy and Winstead, 2014). Regarding the domestic violence, females described the phenomenon from a different perspective, indicating the verbal, physical, and sexual abuse, while males indicated only the verbal abuse. These findings did not support those from investigations of male victims of domestic violence by Drijber et al. (2013); the men were physically as well as the psychologically abused females, and were often an (ex)-partners. Interestingly, in our sample, male victims of stalking were more prone to experience unwanted written communication than females. This confirmed that female stalkers tend to adopt more behaviors that permit them to be connected with their victims (Purcell et al., 2001). The duration of the stalking campaigns was similar in both females and males, with a little longer duration in males; as suggested by Meloy and Boyd (2003), female stalkers are more patient and tough. Male victims are also more prone than females to express anger with their stalkers, though they did not reach threshold values from the psychopathological point of view. This finding confirmed that the expression of this feeling (especially behaviorally) is culturally associated with men (Simon and Nath, 2004).

From the screening for depression, BDI emerged that the discouragement did not prevail for the most part in either male or female victims. However, females tend to amplify, more than males, depression, state, and trait anxiety (in particular the moderate level of trait anxiety). The expression of anxiety symptoms was also seen through the body; indeed, females experienced somatic and cognitive (such as confusion) symptoms. Moreover, in females, there was a higher influence of the victimization in some cognitive aspects that could have had an impact on work efficiency, on the ability to apply social and organizational rules (Acquadro Maran et al., 2014b), medical procedures, and to care the patients (Collins and Long, 2003). The anxiety and the somatization were evident, for example, in the higher percentage of sleep disturbances in female. Even if all symptoms were expressed in both females and males, in males a lack of confidence in their body and of their emotional literacy makes the expression of distress (in each channel, such as emotive and cognitive) more difficult. At the same time, the expression of anxiety presented in women is permitted to become progressively less victimized over time; depression and anxiety permitted the recognition of these symptoms as signs of distress and to intervene to reduce them (Spence-Diehl, 2004).

An interesting finding was in regard to the coping strategies. Victims, both female and male, involved in this investigation confirmed that the coping strategy of moving away was the most used in this population (Acquadro Maran et al., 2017), alone or in association with another. However, our findings suggested that when harassment behavior increased, the number of coping strategies adopted by the victims decreased. An explanation could be in the fatigue resulting from coping with repetitive and intrusive behavior that distress the victim (Davis et al., 2002), leading to exultation. This result was not in accordance with Spitzberg et al. (1998, p. 43) who argued that “the more a person is obsessively pursued, the more this person attempts to cope, and the increased coping is merely a barometer of the stalking and its disruptiveness, rather than a method of effectively diminishing the negative effects of the stalking” (p. 43). According to Davis and Frieze (2000), the link between coping strategies and the stalking campaign needs attention from scholars; the adoption of an appropriate coping strategy (e.g., sought help from colleagues) could determine the stop of the stalking campaign (Kaplan, 2006). In particular, in HCP victims of stalking, the urgency to intervene is linked to the need to limit the consequence of the stalking campaign, in order to be efficient and effective at work.

There were, of course, limitations to this study. First, since the sample was non-randomly selected, the results should be taken with caution and should not be generalized. Moreover, the sexual orientation of the stalker and victim was not investigated; thus, comparisons between heterosexual and non-heterosexual individuals were not made. Studies that had directly assessed the stalking campaign based on sexual orientation found that men were more likely to engage in a stalking campaign at the end of a relationship than women were (Derlega et al., 2011). Furthermore, in this current study, the data on the contradiction between being a HCP victim of stalking/domestic violence and caring for victims of stalking/domestic violence were not collected. Future research should investigate the psychological impact in HCPs who are victims of stalking and domestic violence and caring for victims of stalking and domestic violence. At least one other limitation was in reference to the domestic violence experience. Our work was based on the more well-known categorization of domestic violence [physical, sexual, emotive – see World Health Organization [WHO], 2001 and Garcia-Moreno et al., 2006], but the questionnaire was not tailored to provide detailed information about the experience (for example the economic violence or violence associated with ethnic/religious motives were not investigated). We suggest that future studies examine the experience of domestic violence and its link with sexual orientation and stalking victimization in a more comprehensive way. As argued by Sheridan et al. (2014), the study of those variables could allow a better understanding of the dynamics of the stalking phenomenon, its consequences and for the exploration of the efficacy of coping strategies adopted by victims and their social context.

Despite these limitations, we hope this study offers interesting insights and suggests implications for HCPs and the organization in which they are working. First, attention is generally focused on female victims of domestic violence and stalking. This study highlights, one more time, the importance of considering men as potential victims of domestic violence and stalking (Tarzia et al., 2017). The indication is that HCPs, and the entire health care system, need to improve their ability to recognize the signs of victimization in men, to provide more suitable intervention for individuals (e.g., counseling, Zaccagnino et al., 2017) and the social context (e.g., to protect them and their families). Moreover, the auspice is to consider HCPs not only as providers of care in victims of domestic violence and stalking but also as potential victims themselves. For HCP victims of domestic violence and stalking, due to the nature of their work, it could be more difficult to admit the victimization, particularly when the nature of experiencing violence is intimate. At the same time, the perceived contradiction of being victims and providers of care in victimization cases (Guldimann et al., 2015) could result in a minimization or a denial of the problem (Acquadro Maran et al., 2018). Clearly, such an attitude can be harmful both for the patient/victims and for the HCP victims. In HCP victims, the experience could result in a reluctance to seek support, with a consequently prolonged exposure to the stalking campaign and its effect on well-being. Finally, health care organizations (e.g., hospitals) should contribute to prevent of the phenomenon and should intervene in domestic violence and stalking phenomena. Prevention programs include, for example, information courses on the phenomena (e.g., underlying the prevalence of victimization among HCPs), the risk of victimization (in the general population and in HCP population), and defense strategies (also those offered by the Italian anti-stalking law). Health care organizations should also offer individual measures, such as intervention programs, counseling, and psychological help, to reflect on victimization experiences. Future research should look to replicate – with a larger sample – the current analyses to test the psychological impact of the different forms of domestic violence in HCPs victims of stalking.

## Ethics Statement

This study was carried out in accordance with the recommendations of Italian Law statement about privacy, the Code of Italian Psychologist, and the Ethical Committee of the Univesrità degli Studi di Torino with written informed consent from all subjects. All subjects gave written informed consent in accordance with the Declaration of Helsinki. The protocol was approved by the Hospital administrations and local guarantee committees evaluated, endorsed, and authorized the research, allowing researchers to use the data for scientific purposes.

## Author Contributions

DAM and AV substantially contributed to the conception or design of the work or to the acquisition, analysis, or interpretation of the data for the work. DAM and AV drafted the work or revised it critically for important intellectual content. DAM and AV made the final approval of the version to be published. DAM and AV prepared the agreement to be accountable for all aspects of the work in ensuring that questions related to the accuracy or integrity of any part of the work are appropriately investigated and resolved.

## Conflict of Interest Statement

The authors declare that the research was conducted in the absence of any commercial or financial relationships that could be construed as a potential conflict of interest.
